# Pennsylvania State Veterinary Medical Association

**Published:** 1897-04

**Authors:** 


### PENNSYLVANIA STATE VETERINARY MEDICAL ASSOCIATION.

### OFFICERS AND COMMITTEES.

President, James B. Rayner, West Chester; First Vice-President, M. E. Conard, West Grove; Second Vice-President, J. C. Foelker, Allentown; Third Vice-President, George B. Jobson, Franklin; Recording Secretary, Jacob Helmer, Scranton; Corresponding Secretary, W. L. Rhoads, Lansdowne; Treasurer, John R. Hart, Philadelphia.

Board of Censors: W. H. Ridge, Chairman, Trevose; W. Horace Hoskins, Philadelphia; S. J. J. Harger, Philadelphia; J. C. McNeil, Pittsburg; J. W. Sallade, Pottsville.

Committee on Sanitary Science and Police: J. Curtis Michener, Chairman, Colmar; Francis Bridge, Philadelphia; J. F. Butterfield, South Montrose; J. B. Irons, Erie; S. E. Weber, Lancaster; N. E. Rheinhart, Pottstown; C. C. McLean, Meadville.

Committee on Intelligence and Education: W. H. Ridge, Chairman, Trevose; H. B. Felton, Philadelphia; W. S. Kooker, Philadelphia; Otto G. Noack, Reading; Charles T. Goentner, Bryn Mawr.

Committee on Revision of Constitution and By-laws: Thomas B. Rayner, Chairman, Philadelphia; W. S. Kooker, Philadelphia; W. Horace Hoskins, Philadelphia; Leonard Pearson, Philadelphia; W. H. Ridge, Trevose. *Ex officio*: James B. Rayner, West Chester; W. L. Rhoads, Lansdowne.

REPORT OF THE COMMITTEE ON RESOLUTIONS.

*Resolved*, That this Association indorse such measures taken by the Pennsylvania State Board of Veterinary Medical Examiners as will lead to the establishment of the dehorning of cattle as a surgical operation to be practised only by qualified veterinarians.

WHEREAS, During the past year many of our members have been sorely afflicted by trials and losses unusual in number and severe in character, that we have felt that we should in our sympathy for our fellow-members take some recognition as a token of our esteem and fellowship. We specially refer to the affliction of our fellow-members, Drs. Thomas B. Rayner, J. C. Foelker, and R. G. Webster, in the loss of members of their respective families, and to the serious illness of our fellow-members, Drs. James B. Rayner and S. E. Weber. To all of these we extend our sincere sympathy, and trust that these afflictions may be tempered in their severity to our fellow-members.

WHEREAS, This Association regrets extremely to hear of the death during the past year of William Tag, Ph.G., V.M.D., of Philadelphia, graduate of the Veterinary Department of the University of Pennsylvania, class of '91, who was for a considerable time a member of this Association and was respected by those of us who knew him best; be it further

*Resolved*, That these resolutions be spread upon the minutes of this Association, under a suspension of rules governing the order of business, and that a copy of the same be sent to the widow of the deceased.

WHEREAS, So many recommendations have been made by our President and members involving changes in the By-laws, and in that there are many points in which the present printed laws do not seem to cover clearly, we recommend the appointment of a Committee on Revision of the Constitution and By-laws.

WHEREAS, The State Board of Health of Pennsylvania is engaged in the important work of improving the sanitary conditions under which the inhabitants of our Commonwealth are obliged to live; and

WHEREAS, The results which have thus far been achieved by said board are of the greatest value to all citizens of the State and conducted in an economical but at the same time profitable and valuable manner, which deserves the support and confidence of the public, and the expense of their work has never been in proportion to the good that has resulted from it; and

WHEREAS, This condition results principally from the indefatigable energy of its members, and particularly of its Secretary, Dr. Benjamin Lee; and

WHEREAS, It is not proper for the State to place upon its servants duties that are so necessary and arduous as those that fall to the Secretary of the State Board of Health without making proper compensation therefor; it is hereby

*Resolved*, That the Legislature of the State of Pennsylvania is respectfully petitioned to increase the appropriation at the disposal of the State Board of Health to such an amount that its work can be carried on more extensively and readily; and be it further

*Resolved*, That in said appropriation provision should be made for an increase in the salary of the Secretary, Dr. Benjamin Lee, to an amount commensurate with the duties he has to perform.

WHEREAS, The subject of horseshoeing has been much neglected in this country, and no schools for the thorough and scientific education of horseshoers have ever been established in the United States, and there is a great dearth of reliable literature on this subject; and

WHEREAS, The matter of horseshoeing is of the greatest importance to the well-being of horses and their economical use; and

WHEREAS, It is desirable that knowledge of this important subject should be distributed to a greater extent; be it

*Resolved*, That the State Veterinary Medical Association of Pennsylvania hereby pledges its support to any movement that has for its object the dissemination of practical and reliable information in reference to the shoeing of horses; and it regrets that schools have not heretofore been established wherein horseshoers could be given a thorough knowledge of their trade, and hope that this defect will soon be remedied by the further development of movements already inaugurated.

WHEREAS, The live-stock industry of Pennsylvania is of great magnitude and is so indispensable to agriculture and to the inhabitants of the State at large; and

WHEREAS, It is at this time harassed and drained by a number of transmissible diseases, most of which are or will be preventable; and

WHEREAS, It is not possible for the State authorities to deal with sufficient system or accuracy with all of the questions connected with these diseases, on account of absence of full information regarding them; and

WHEREAS, Such essential information can only be obtained by laboratory and field experiments and investigations conducted on an adequate scale; it is hereby

*Resolved*, That the Legislature of Pennsylvania is requested to provide means for the inauguration and continuation of studies of the diseases afflicting the domestic animals of this Commonwealth, in accordance with a plan comprehensive enough to allow accurate conclusions to be reached that may safely guide veterinarians, live-stock owners, and the State authorities in dealing with these momentous questions; and be it further

*Resolved*, That a copy of these resolutions shall be transmitted by the Secretary to the President of the Senate and the Speaker of the House and to the Chairman of the Committee on Agriculture of the Senate and of the House of Representatives, and to the Secretary of the State Live-stock Sanitary Board.

WHEREAS, As it was largely through the efforts of the Pennsylvania State Veterinary Medical Association that the law organizing the State Live-stock Sanitary Board was passed by the last Legislature; and

WHEREAS, The veterinarians of the State have been greatly interested in and have carefully observed the policy and progress of this board; and

WHEREAS, Its work has been marked by efficiency, liberality, and conservatism, and has succeeded to a remarkable degree in disarming the critics who objected to its organization; and

WHEREAS, This desirable effect has resulted largely through the efforts of the State Veterinarian, Dr. Leonard Pearson; be it

*Resolved*, That the thanks of this organization be and are hereby extended to the Governor, Hon. Daniel H. Hastings, for appointing the present State Veterinarian in accordance with the expressed wishes of this Association; and be it further

*Resolved*, That the State Live-stock Sanitary Board is hereby congratulated upon formulating and putting into effect such practical and valuable measures for the suppression of the diseases of animals and methods that are in full accord with the requirements of the situation as far as they can be met with at this time; and be it further

*Resolved*, That a copy of these resolutions shall be sent to the Governor and to each of the other members of the State Live-stock Sanitary Board.

Committee on Resolutions: W. Horace Hoskins, Chairman; B. F. Senseman, and M. E. Conard.

### AFTERMATH OF THE CONVENTION.

Ex-President Ridge may well feel a justifiable pride in the year's work of the Association.

One of the Association's members was not at his best, owing to the suffering incidental to cutting of a wisdom tooth, which promises much in the way of increased activity and Association work.

President James B. Rayner well deserved the honor conferred upon him, and we are assured that he will have earnest support from every member.

The Chairman of the Local Committee of Arrangements looked aghast on the first day, when he had provided luncheon for forty, and sixty guests presented themselves; he proved equal to the occasion.

The Philadelphia newspapers were nearly all represented and gave extended accounts of the Convention's work—especially so the *Ledger's* representative, Mrs. E. S. Starr—for all of which the members were more than thankful.

Many were the sympathetic expressions heard on all sides for the unusual number of our members upon whom sore trials and illness had fallen during the past year.

Franklin will make a royal place for our meeting in September next. With our Third Vice-President Mayor of that hustling city, safety and protection are at least assured us.

Great was the interest shown in the Convention's work by the students of the Veterinary Department of the University of Pennsylvania, and they are to be in great measure the future supporters of our Association. Over thirty of them were there at the several sessions of the Convention.

In Secretary Rhoads the Association will find a hustling, up-to-date Secretary. His paper, "Why?" was pungent, timely, and will do much good in Association circles when read.

Secretary McAnulty, of the State Horseshoers' Association, was very much delighted by the passage of the resolutions giving encouragement to the efforts to establish schools of instruction for their craft.

The old guard was not there; sickness and death have touched keenly our members Rayner and Foelker.

After a trial of strength for Doylestown and Williamsport, it was made unanimous to go to Franklin.

Many were the inquiries as to the whereabouts of Chairman Zuill, of the Committee on Intelligence and Education. For a year not one line has been forthcoming from this committee. Referred to Secretary Rhoads—Why?

Somebody side-tracked a little boom of one of Philadelphia's candidates for First Vice-President. Well, it has not gone wrong in falling upon the shoulders of member Conard.

Where does the City of Brotherly Love come in among the elective officers—oh, yes, it has the Treasurer, the "fellow who sits on the chist," and a good, sound, safe, iron-clad officer he is.

The County Secretaries were fully alive to their work this year and sent in many good reports.

The programme was loaded. Can we not have the same at Franklin?

No less than four of the six veterinarians employed by our city were at the Convention. We were glad to note this renewed interest.

Delegate Adams struck the key-note when he said there was only a place for *one* good, strong local association in Philadelphia.

Are our members in the Lehigh Valley losing their interest? Secretary Helmer will now have to look up our supporters in that district. He will make an ideal officer.

Secretary *pro tem.* Kooker found it a little hard to get back in harness again, but the Association never had a more efficient one than during his term of office, and it never did more work throughout the State.

Now we propose that the trip to Franklin in September shall include the ladies. Let there be no stay-at-homes.

Outgoing Secretaries Benner and Allen have been very efficient officers and well deserved the thanks of the members for the able and conscientious discharge of their duties.

There seems to be no limit of work for the Keystone State Association; each year finds it increasing in volume and worth. So much for good executive officers.

The Association badly needs a press committee to better aid the proper publicity of our work. Referred to the Committee on Revision of Constitution and By-laws.

Our visitors from New Jersey and Delaware added pleasure and interest to the meeting. There should be others.

Should not the Board of Trustees be increased to seven? Ask the Committee on Revision. We now have about 130 members, and the proposition to have it made up of retiring presidents may quickly lose some of the most valuable of our members.

One of our regular attendants from Bucks County was denied the pleasure of being there, but there was an added pleasure and responsibility at home —a daughter.

One of the popular members given to recitations at the luncheon is now a devoted student of the vocal art, and hopes by next year's meeting to entertain the members with a song.

